# Performance and Value of IFN-Lambda3 and IFN-Lambda4 Genotyping in Patients with Chronic Hepatitis C (CHC) Genotype 2/3 in a Real World Setting

**DOI:** 10.1371/journal.pone.0145622

**Published:** 2015-12-23

**Authors:** Steffen B. Wiegand, Benjamin Heidrich, Simone Susser, Magdalena Rogalska-Taranta, Jörg Petersen, Klaus H. W. Böker, Natalia Grigorian, Ralph Link, Uwe Naumann, Christine John, Stefan Lueth, Peter Malfertheiner, Michael P. Manns, Heiner Wedemeyer, Christoph Sarrazin, Markus Cornberg

**Affiliations:** 1 Department of Gastroenterology, Hepatology and Endocrinology, Hannover Medical School, Hannover, Germany; 2 German Liver Foundation, HepNet Study-House, Hannover and Frankfurt, Germany; 3 Department of Internal Medicine I, University of Frankfurt, Frankfurt, Germany; 4 German Center for Infectious Diesease (DZIF), Hannover-Braunschweig, Germany; 5 IFI Institute for Interdisciplinary Medicine, Asklepios Klinik St Georg, Hamburg, Germany; 6 Medical Practice, Hannover, Germany; 7 Department of Internal Medicine II, University of Saarland, Saarland, Germany; 8 Department of Internal Medicine, Clinic for Internal Medicine St. Josefs hospital, Offenburg, Germany; 9 Center for Addiction-Medicine, Hepatology and HIV, Praxiszentrum Kaiserdamm, Berlin, Germany; 10 Medical Practice, Berlin, Germany; 11 Department of Internal Medicine, University of Hamburg, Hamburg, Germany; 12 Department of Gastroenterology, Hepatology and Endocrinology, University of Magdeburg, Magdeburg, Germany; National Taiwan University Hospital, TAIWAN

## Abstract

**Background:**

SNPs near the interferon lambda (IFNL) 3 gene are predictors for sustained virological response (SVR) in patients with chronic hepatitis C genotype (GT) 1. In addition, a dinucleotide frame shift in ss469415590 was described, which generates IFNL4. In this study, we compared the role of IFNL4 variants with IFNL3-(rs12979860) and IFNL3-(rs8099917) on response to pegylated (PEG)-IFN and Ribavirin (RBV) in patients with chronic hepatitis C GT2/3.

**Methods:**

We recruited 1006 patients with chronic hepatitis C and GT2/3 in a large German registry. A treatment with PEG-IFN and Ribavirin was started by 959 patients. We performed genotyping of IFNL3 (rs12979860, n = 726; rs8099917, n = 687) and of IFNL4 (ss469415590; n = 631).

**Results:**

Both preferable IFNL3 genotypes were associated with RVR (both p<0.0001) rather than with SVR (rs12979860: p = 0.251; rs8099917: p = 0.447). Only RVR was linked to SVR in univariate and multivariate analyzes (both p<0.001). Concordance of genotyping in patients with available serum samples and EDTA blood samples (n = 259) was more than 96% for both IFNL3 SNPs. IFNL3-(rs12979860) correlated with IFNL4: 99.2% of patients with IFNL3-(rs12979860)-CC were IFNL4-(ss469415590)-TT/TT. IFNL3-(rs12979860)-CT was linked with IFNL4-(ss469415590)-TT/ΔG (98.0%) and IFNL3-(rs12979860)-TT was associated with IFNL4-(ss469415590)-ΔG/ΔG (97.6%).

**Conclusion:**

IFNL3 genotyping from serum was highly efficient and can be used as an alternative if EDTA whole blood is not available. In Caucasian GT2/3 patients genotyping for INFL4-(ss469415590) does not lead to additional information for the decision-making process. Importantly, IFNL3 SNPs were not associated with SVR but with RVR. Even in the era of new direct acting antiviral (DAA) therapies, IFNL3 testing may therefore still be considered for naïve GT2/3 patients to decide if dual Peg-IFN/RBV therapy is an option in resource limited regions.

## Introduction

World-wide 64–103 million people are chronically infected with the hepatitis C virus (HCV) [1]. There are seven HCV genotypes, which show different distributions around the world. Genotype 2 is frequent in some parts of Asia. Genotype 3 is very frequent in South-East Asia and accounts for around one third of infections in Europe [2]. HCV genotypes have influence on the natural course of chronic hepatitis, i.e. genotype 2 is associated with ALT flares while genotype 3 infected patients show faster disease progression and higher mortality [3,4]. In addition, different genotypes require different treatment concepts, now and in the past.

For decades IFN in combination with RBV was the standard of care chronic hepatitis C. While patients with genotype 1 were treated for 48 weeks resulting in 50% SVR, patients with genotypes 2 and 3 achieved up to 90% SVR with 24 weeks therapy [5,6]. Similar to HCV genotype 1, IFN based therapy can be individualized based on viral and host factors. For example, patients with high viral load at baseline achieved significantly less often SVR [7]. Host factors such as age, gender, stage of fibrosis, origin and body mass index affect treatment outcome in interferon based therapy regimes [8]. In recent years genome-wide association studies (GWAS) revealed several genetic polymorphisms (rs12979860; rs8099917; etc.) in the interferon lambda 3 (IFNL3) gene region (also known as IL28B), which are associated with SVR rates in patients with HCV genotype 1 undergoing combination therapy with pegylated interferon and ribavirin (PegIFN/RBV) [9–11].

In the last few years several new DAA have been approved for the treatment of hepatitis C. DAA combinations with or without IFN showed improved SVR rates up to 95%, especially in genotype 1 patients with shorter treatment duration [12]. However, in 2015 many of the available DAAs have some limitations in efficacy in genotype 2 and especially in genotype 3. In addition, novel DAA are not available in all parts of the world at the same time. Thus, IFN based therapies; especially for genotype 3 are still an important therapeutic concept [13]. So far, the data regarding an association of different IFNL3 genotypes with SVR in HCV genotype 2 and 3 patients is still controversial [14–19]. One study by Eslam et al. showed an associations between IFLN3 genotypes and SVR in patients with HCV genotype 2 and 3 [18], while other studies have demonstrated less clear results [14–17,19]. One reason for the discrepancy may be due to small sample sizes used in some of these trials and the heterogeneity of subgroups analyzed [14,17,18,20]. Recently, IFNL4 a new variant in the CpG region upstream of IFNL3 (IL28b) was observed which showed also strong association with HCV clearance [21]. Susser et al., showed that in genotype 3 infected patients, best SVR prediction was based on IFNL4 and not on IFNL3 genotype [22]. However, also in this study the number of genotype 3 patients was lower than 200. Therefore, the aim of this study was to analyze the association of IFNL3 and IFNL4 genotypes with therapy outcome in more than 600 treatment-naïve patients infected with genotypes 2 and 3 treated with pegylated interferon alfa-2b and ribavirin within a large multi-center nationwide prospective registry.

## Material and Methods

### Patient population

**Fig 1:**
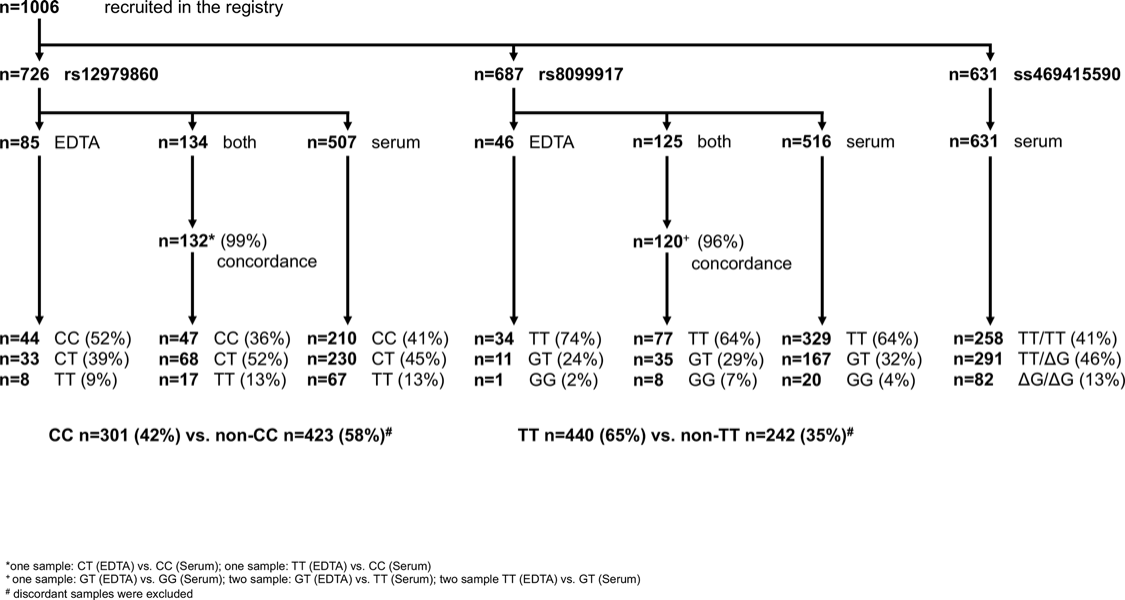
Flow chart of patients with available IFNL3 and IFN-L4 genotype.

Overall, 152 centers participated in this prospective German nationwide multicenter registry and 1006 patients were recruited between June 2008 and December 2012. Eligibility criteria for the registry were age ≥18, HCV genotype 2 or 3, detectable HCV RNA (>15 IU/mL) and positivity of anti-HCV antibodies as well as no history of antiviral therapy (Fig 1). Overall, 959 started treatment with PEG-IFN and Ribavirin with 114 being HCV genotype 2 and 567 being HCV genotype 3 with available data on IFNL3 and 4. Cirrhosis was present in 7% of the overall population. Detailed in- and exclusion criteria and baseline characteristics can be found in the supplementary materials (S1 Fig and S1 Table).

### Interferon Lambda 3 (IFNL3) genotyping In EDTA-blood

IFNL3 (formerly IL28B) single-nucleotide polymorphism rs12979860 and rs8099917 genotyping was performed using real-time polymerase chain reaction and melting curve analysis in the Light Cycler 480 II System (Roche, Mannheim, Germany). DNA was extracted from EDTA-blood samples using the DNeasy purification Kit (QIAGEN, Hilden, Germany). Primers and hybridization probes were purchased from TIB MOLBIOL (Berlin, Germany).

### Genotyping of IFNL3 (IL28B) and IFN-L4 in serum

Genotypes of rs8099917 (*IFNL3*), rs12979860 (*IFNL3*) and ss469415590 (*IFN-L4*) were determined using an inventoried TaqMan^®^ SNP Genotyping assay (Life Technologies GmbH, Darmstadt, Germany) for rs8099917 and custom-designed TaqMan^®^ SNP Genotyping assays (Life Technologies GmbH, Darmstadt, Germany) for rs12979860 and ss469415590.

All reactions were set up with 1μL of isolated gDNA and TaqMan^®^ Genotyping Master Mix (Life Technologies GmbH, Darmstadt, Germany). The genotyping ran on a StepOnePlus^TM^ Real Time PCR System (Life Technologies GmbH, Darmstadt, Germany). Genotyping was performed at the Biomedical Research Laboratory of Medical Clinic 1, Goethe-University Hospital, Frankfurt, Germany.

### HCV RNA quantification

HCV RNA quantification was performed at baseline (BL), W4, -12, end of treatment and 24 weeks after cessation of therapy. Quantification was done locally with different assays and thresholds [13].

### Response definitions

HCV-RNA below 15 IU/mL at week 4 of treatment with pegylated interferon and ribavirin was defined as rapid virological response (RVR) according to the German Guidelines and based on the analysis by Sarrazin et al., [23,24]. A decrease of more than 2 log10 from baseline in HCV RNA or HCV RNA negativity at week 12 was defined early virological response (EVR); end of treatment response (EOT) and sustained virological (SVR) were defined as HCV-RNA below detection limit 24 weeks after the end of treatment. In the intention-to-treat (ITT) analysis all patients with at least one dose of PegIFN and RBV were included. Missing results for RVR and SVR were considered as negative results. For the per-protocol (PP) analysis, patients treated for at least 12 weeks and with available results at week 4 and/or 24 weeks after end of therapy were considered for each evaluation.

### Laboratory tests

Hematological and biochemical parameters were assessed locally at baseline. Alanine aminotransferase (ALT), aspartate aminotransferase (AST), gamma-glutamyltransferase (GGT) and alkaline phosphatase (ALP) as well as bilirubin, creatinine and albumin were part of the biochemical work-up. Hematological parameters included platelet counts.

### Definition of liver cirrhosis

Non-invasive methods like ultrasound, FibroScan® or biochemical results and liver histology were used for defining presence or absence of cirrhosis. F4 in Metavir or F5-6 in ISHAK in liver biopsies was considered as cirrhosis [25,26]. Diagnosis of cirrhosis in ultrasound was based on assessment of the local physician. Same holds true for steatosis. Liver stiffness ≥12.5kPa was considered as cirrhosis [27]. Patients with at least two of the following criteria: AST/ALT ratio >1 [28], bilirubin >1,5ULN, platelets <100/nL and albumin <35g/L fulfilled biochemical assessment of cirrhosis. If one of the definitions above was met, presence of cirrhosis was considered.

### Statistical analysis

GraphPad Prism 5 (GraphPad Software, Inc., La Jolla, CA, USA) was used for graphic design. Statistical analysis was performed with SPSS, version 22 (2013, SPSS, Munich, Germany). Quantitative values are indicated in median or mean and statistical differences were assessed by using Student’s t test. Qualitative data was analyzed by using Chi square test. Differences were considered significant at p<0.05. An online Tool (http://www.oege.org/software/hwe-mr-calc.shtml) for calculating the Hardy-Weinberg equilibrium based on Rodriguez publication was used [29].

### Ethical approval

The ethics committee of Hannover Medical School, Medical University of Leipzig, Regensburg, Cologne, Essen, Bonn, Bochum, Münster, Rostock, Halle-Wittenberg, Würzburg and the State Chamber for Physicians of Thuringia, Saxony, Saxony-Anhalt, North-Rhine, Lippe-Westphalia, Saarland and Bremen approved the registry. Each patient signed a written informed consent form. The registry has been performed according to the current World Medical Association Declaration of Helsinki. The procedures have been approved by the local ethics committee of the Hannover Medical School (Vote No. 3860) and are in line with German law.

## Results

### IFNL3 and IFNL4 genotyping

Overall, 726 out of 1006 (72%) individuals have been genotyped for IFNL3 rs12979860 and 687 samples were tested for IFNL3 rs8099917 (Fig 1). Additionally, 631 samples could be genotyped for IFN-L4 ss469415590. The genotype distribution of the IFNL4 double nucleotide polymorphism and the two IFNL3 SNPs was in Hardy-Weinberg equilibrium. The overall genotype distribution of IFNL3 rs8099917 TT, GT, and GG was 64.3%, 31.4%, and 4.2% and the distribution of rs12979860 CC, CT, and TT was 41.5%, 45.7%, and 12.8%, respectively. For IFNL4 ss469415590 TT/TT, TT/ ΔG, and ΔG/ΔG were seen in 40.9%, 46.1%, and 13.0% of patients, respectively.

### Concordance in IFNL3 genotyping in EDTA whole blood and serum

Testing for IFNL3 rs12979860, IFNL3 rs8099917 and IFNL4 ss469415590 was not scheduled in the initiation phase of this registry and was performed with available stored EDTA whole blood samples and/ or serum samples as illustrated in Fig 1.

Overall, in almost all samples concordant results between serum and EDTA whole blood samples were observed in IFNL3 rs12979860 and rs8099917 genotypes with concordance rates of 99% (132 out of 134) and 96% (120 out of 125), respectively.

In those two cases with discordance for rs12979860 genotype one patient had CT in EDTA whole blood and CC in serum and the other had TT in EDTA whole blood and CC in serum (Fig 1). For genotyping of rs8099917 discordant results were observed in five cases (1 sample: GT (EDTA) vs. GG (serum); 2 samples: GT (EDTA) vs. TT (serum); 2 samples: TT (EDTA) vs. GT (serum)) (Fig 1).

### IFLN4 ss469415590 in Caucasian patients with HCV G2/3

In addition, serum samples of 631 patients were tested for IFLN4 genotype. The overall genotype distribution of IFNL4 ss469415590 TT/TT, TT/ΔG, and ΔG/ΔG was 40.9%, 46.1%, and 13.0%. We observed a very high correlation between IFNL3 (rs12979860) and IFN-L4 (ss469415590) (r = 0.9777; p<0.0001). IFNL3 (rs12979860) CC was associated with IFN-L4 (ss469415590) TT/TT in 99.2% of patients. IFNL3 (rs12979860) CT was linked with IFN-L4 (ss469415590) TT/ΔG (98.0%) and IFNL3 (rs12979860) TT was associated with IFN-L4 (ss469415590) ΔG/ΔG (97.6%).

### Treatment response in correlation with IFNL3 and IFLN4 genotypes

**Fig 2:**
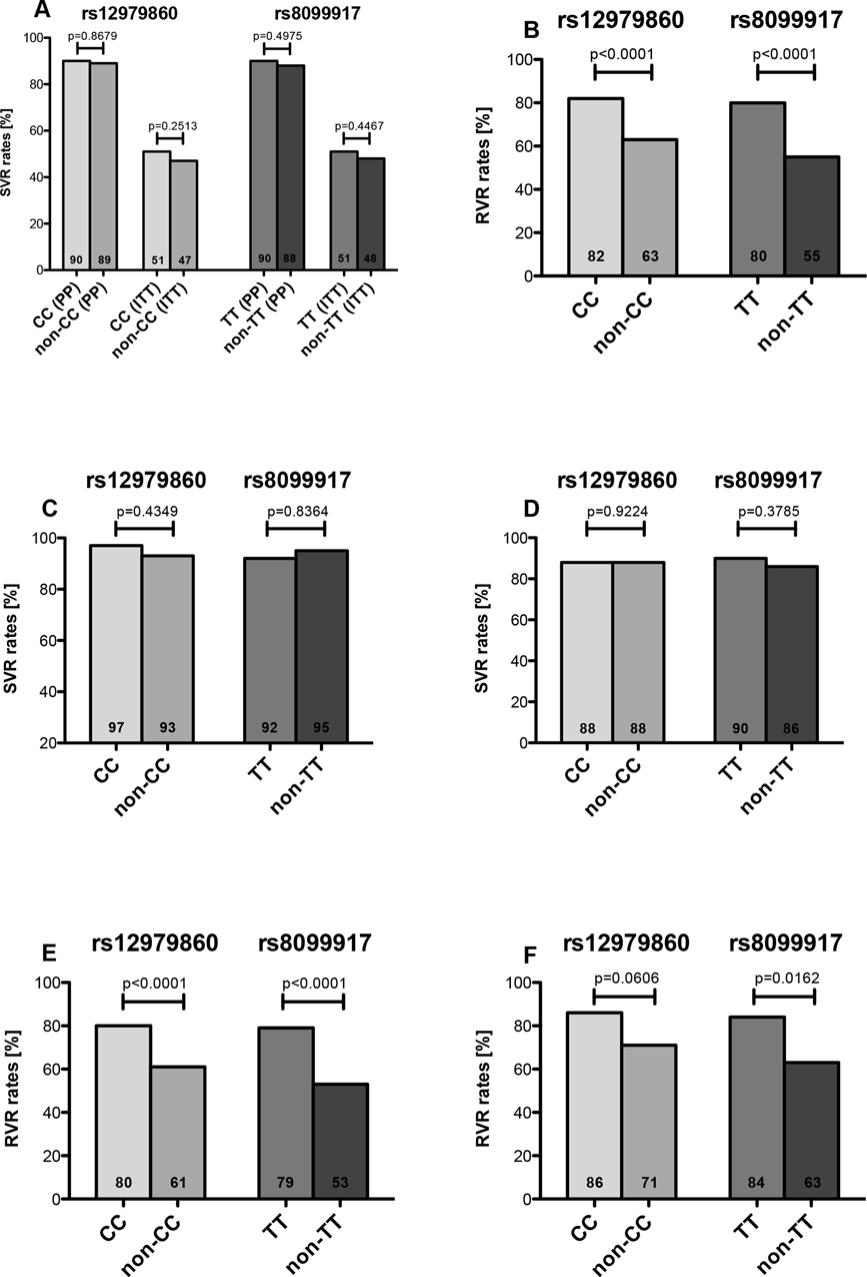
SVR and RVR rates according to INF-L3 and HCV genotype. **(A)** SVR rates in relation to IFNL3 genotype (PP analysis). (B) RVR rates in relation to IFNL3 genotype (PP analysis). (C) SVR rates in relation to IFNL3 genotype in HCV genotype 2 patients. (D) SVR rates in relation to IFNL3 genotype in HCV genotype 3 patients. (E) RVR rates in relation to IFNL3 genotype in HCV genotype 3 patients. (F) RVR rates in relation to IFNL3 genotype in HCV genotype 2 patients.

Due to the high association between IFNL3 (rs12979860) and IFN-L4 (ss469415590) we only used IFNL3 SNPs for further analyses. There was no statistical difference considering SVR rates in ITT as well as PP analysis in different SNPs (rs12979860 and rs8099917) of IFNL3 (Table 1 and Fig 2A). The so-called preferable genotypes CC-rs12979860 and TT- rs8099917 showed higher RVR rates compared to the non-preferable ones (82% vs. 63%; p<0.0001 and 80% vs. 55%; p<0.0001) (Fig 2B).

**Table 1:** SVR rates in relation to IFLN3 genotype.

Analysis	rs12979860			p-value*	rs8099917			p-value*
	CC	CT	TT		TT	GT	GG	
**ITT**	51.2%	49.2%	37.9%	0.09	51.1%	50.0%	33.3%	0.20
**PP**	89.6%	89.2%	89.2%	0.99	90.4%	88.9%	81.8%	0.62
	**CC**	**Non-CC**			**TT**	**Non-TT**		
**ITT**	51.2%	46.8%		0.25	51.1%	48.1%		0.45
**PP**	89.6%	89.2%		0.87	90.4%	88.3%		0.50

* Chi square test

During this study period we observed 32 relapses and only 5 breakthroughs in the per-protocol analysis. The majority of patients with relapse were HCV genotype 3 (n = 27; 84%) and had more often the favorable genotype TT-rs8099917 rather than non-TT-rs8099917 (n = 16 (62%) vs. n = 10 (38%)). In contrast, the favorable genotype CC-rs12979860 was observed in the minority of patients with relapse (n = 11 (39%) vs. n = 17 (61%)).

### Correlation of IFNL3 genotypes and treatment response in different HCV genotypes

The overall SVR was high (91%) for both HCV genotypes. However, patients infected with HCV genotype 2 had significantly higher SVR rates than patients with HCV genotype 3 (96% vs. 90%; p = 0.0479). Week 4 responses were comparable to SVR results but without being statistical significant (78% vs. 70%; p = 0.0520).

The preferable genotype distribution of both SNPs was similar in GT2 and GT3 (rs12979860: 44% vs. 41%; p = 0.5563 and rs8099917: 66% vs. 64%; p = 0.6449) (Table 2). Regarding SVR rates, there were no statistical significant differences between preferable and non-preferable IFNL3 genotype in HCV genotype 2 and 3 (Fig 2C and 2D). Patients with HCV genotype 3 had significantly higher RVR rates if both preferable IFNL3 SNPs were present (CC 80% vs. non-CC 61%; p<0.0001 and TT 79% vs. Non-TT 53%; p<0.0001) (Fig 2E). In patients with HCV genotype 2 only IFNL3 rs8099917 and not rs12979860 showed significant differences in RVR rates (TT: 84% vs. non-TT: 63%; p = 0.0162; CC: 86% vs. non CC: 71%; p = 0.0606) (Fig 2F).

**Table 2:** IFNL3 genotype distribution in HCV genotype 2 and 3.

	rs12979860		p-value	rs8099917		p-value
	CC	non-CC		TT	non-TT	
	n = 301	n = 425		n = 442	n = 245	
In GT2 (%)	44	56	0.5141	66	34	0.6449
In GT3 (%)	41	59	0.5141	64	36	0.6449

### Predictor of treatment responses

Pretreatment clinical variables and on-treatment responses were analyzed considering association to SVR. Only younger age, stage of liver fibrosis, HCV genotype and RVR were linked with SVR-PP in a univariate analysis. All parameters with a p-value ≤0.05 were considered for multivariate analysis. Only RVR was independently associated with SVR-PP in multivariate analysis (p<0.001) (Table 3). A retrospective power analysis showed a requirement of n>383 for rs12979860 genotype and n>391 for rs8099917 genotype in order to have 90% power to detect an odds ratio of 2 with a p-value of 0.05. SVR-ITT analyses fulfilled these criteria and in addition revealed an effect of gender and BMI in the univariate analysis, but only RVR remained independently correlated with SVR (Table 3).

**Table 3:** Univariate and multivariate analysis of pretreament and on treatment parameters considering SVR.

	SVR-PP	Univariate analysis	Multivariate analysis	SVR-ITT	Univariate analysis	Multivariate analysis
	n = (%)	p =	p =	n = (%)	p =	p =
Age<40	167 (96)	0.050	0.600	167 (45)	0.026	0.783
Age>40	298 (88)	0.050	0.600	298 (53)	0.600	0.600
Male	279 (89)	0.069		279 (45)	<0.001	0.170
Female	190 (94)	0.069		190 (59)	0.069	0.170
BMI>27	162 (92)	0.716		162 (55)	0.022	0.978
BMI<27	297 (91)	0.716		297 (47)	0.022	0.978
Cirrhosis	25 (74)	<0.001	0.435	25 (33)	0.005	0.580
Non-cirrhosis	444 (92)	<0.001	0.435	444 (50)	0.022	0.580
Steatosis	128 (90)	0.359		128 (57)	0.103	
Non-steatosis	281 (92)	0.359		281 (50)	0.103	
Genotype 2	99 (96)	0.048	0.217	99 (61)	<0.001	0.147
Genotype 3	366 (90)	0.048	0.217	366 (48)	<0.001	0.147
BL <800,000 IU/mL	241 (92)	0.243		241 (48)	0.603	
BL >800,000 IU/mL	218 (89)	0.243		218 (50)	0.603	
CC- rs12979860	147 (90)	0.893		147 (51)	0.251	
Non-CC- rs12979860	190 (89)	0.893		190 (47)	0.251	
TT- rs8099917	217 (90)	0.590		217 (51)	0.447	
Non-TT-rs8099917	113 (88)	0.590		113 (48)	0.447	
RVR (PP or ITT)	340 (95)	<0.001	<0.001	340 (59)	<0.001	<0.001
Non-RVR (PP or ITT)	80 (77)	<0.001	<0.001	130 (34)	<0.001	<0.001

### Association between viral load and IFNL3 genotypes

Patients with HCV genotype 2 and 3 had similar median levels of HCV RNA at baseline (6.2±1.6 log10 IU/mL vs. 5.7±1.4 log10 IU/mL; p = 0.1838). However, in HCV genotype 2 the frequency of patients with high viral load above 800,000 IU/mL was significantly higher compared to genotype 3 patients (62% vs. 44%; p<0.0001).

**Fig 3:**
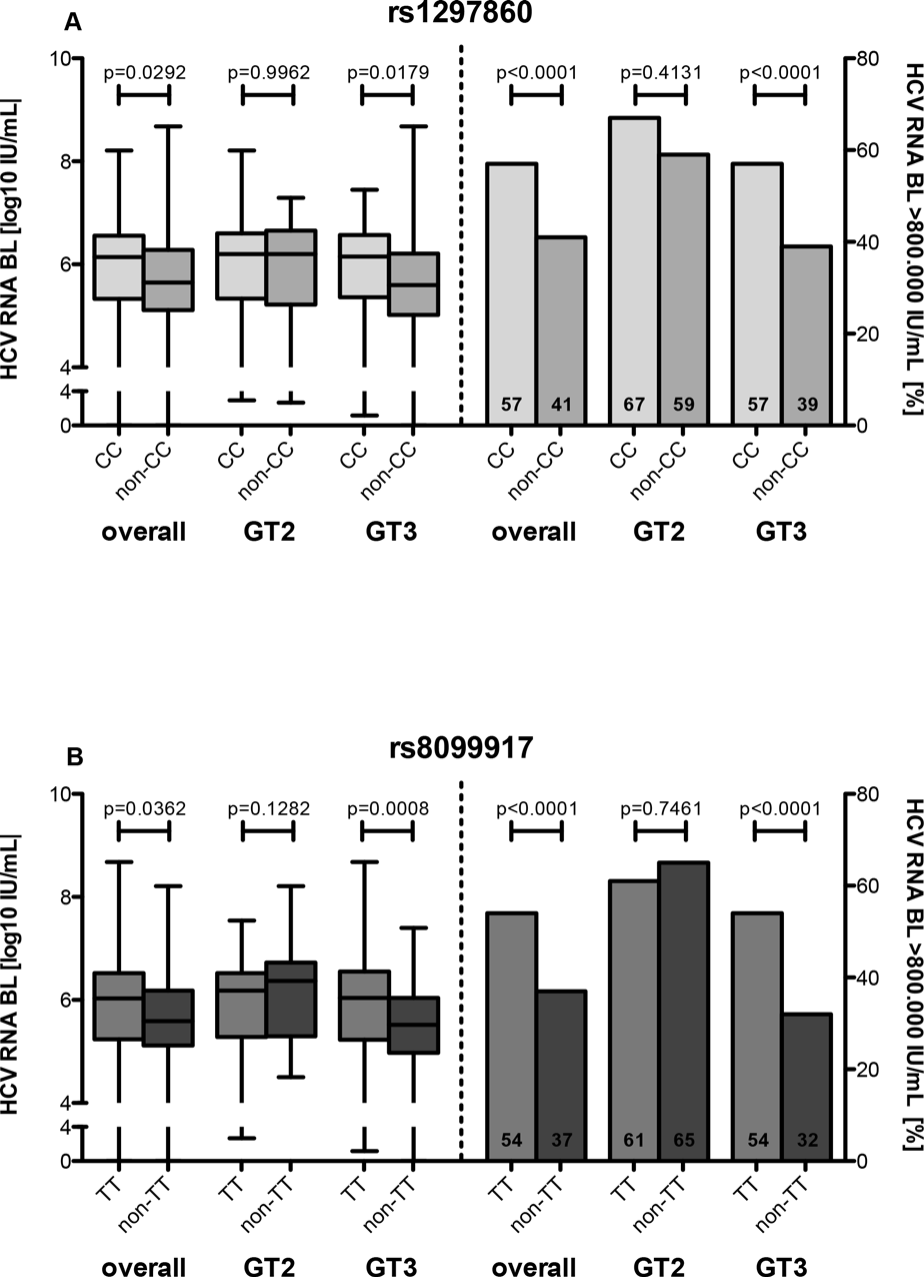
HCV RNA levels at baseline according to IFNL3 genotypes. (A) HCV RNA levels at baseline for patients with favourable and non-favorable rs1297860 genotype. (B) HCV RNA levels at baseline for patients with favourable and non-favorable rs8099917 genotype.

Patients with CC genotype (rs12979860) had significantly higher median baseline HCV RNA levels compared with non-CC genotype (6.1±1.2 log10 IU/mL vs. 5.6±1.0 log10 IU/mL; p = 0.0292) and had more often high viral load (57% vs. 41%; p<0.0001) (Fig 3A). Interestingly, these differences seen in the overall population could be observed only in HCV genotype 3 patients. The association between rs8099917 genotypes and HCV viral load revealed similar results (TT: 6.0±1.2 log10 IU/mL vs. non-TT: 5.6±1.0 log10 IU/mL; p = 0.0362) (Fig 3B).

### Week 4 response in HCV genotype 2/3 patients according to viral load and IFNL3 genotypes

**Fig 4:**
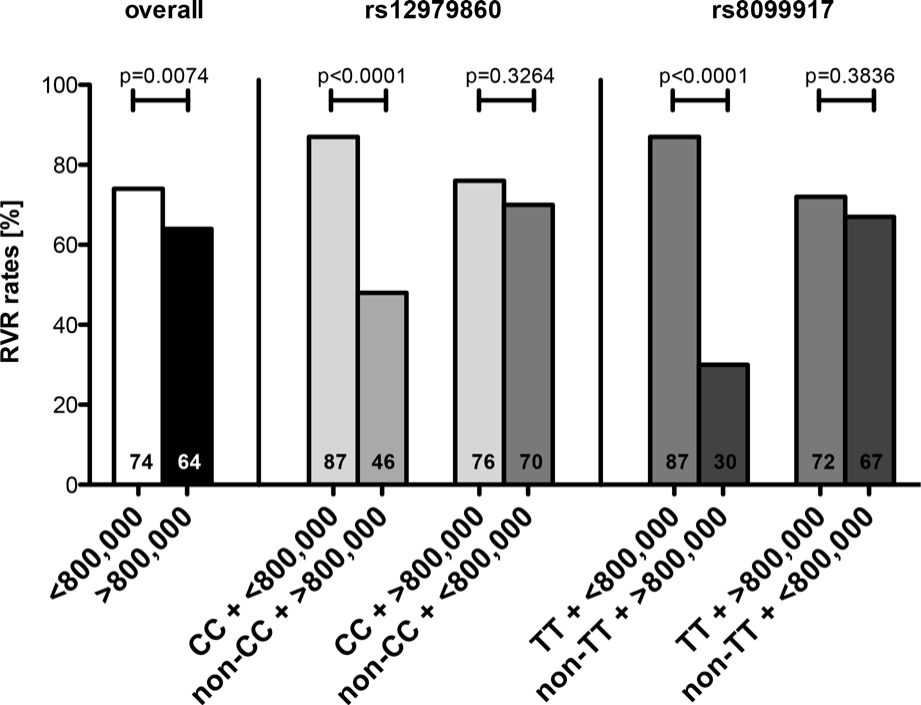
RVR in HCV genotype 3 according to baseline viral load in relation to IFNL3 genotype.

Patients with genotype 3 and low viral load, defined as HCV RNA below 800,000 IU/mL at baseline, achieved more often RVR (74% vs 64%; p = 0.0074) (Fig 4). Low viral load at baseline and each favorable genotype were associated with significantly higher RVR rates. Individuals with favorable IFNL3 genotype and low viral load at baseline compared to patients with non-favorable IFNL3 genotype and high viral load at baseline, had more pronounced differences in RVR rates (rs12979860: 87% vs. 48%; p<0.0001 and rs8099917: 87% vs. 30%; p<0.0001) (Fig 4). In patients with beneficial IFNL3 genotype but high viral load RVR rates were comparable to individuals with non-beneficial genotype but low viral load, (rs12979860: 76% vs. 70%; p = 0.3264 and rs8099917: 72% vs. 67%; p = 0.3836) (Fig 4).

Data with those virological and IFNL3 configuration considering SVR rates are located in the supporting information (S1A Fig).

The same analyses, considering RVR and SVR rates, have been performed for G2 patients with similar results in parts (S1B and S1C Fig). However, the numbers of patients in the subgroups were low.

## Discussion

In this large cohort of more than 600 patients chronically infected with HCV genotype 2 or 3 and available IFNL3 data we could show (i) association of IFNL3 and IFNL4 with RVR rather than SVR, (ii) strong association between IFNL4 ss469415590 and IFNL3 rs12979860 genotypes (iii) and high concordance between IFNL3 genotypes determined in serum and in EDTA-blood.

The role for IFNL3 or IFNL4 testing for patients with genotype 2 and 3 still remains controversial. Many studies have shown an association of IFL3 and RVR but not SVR ([14–19]). However, recently, Eslam et al. [18] demonstrated a high association of both preferable SNPs with SVR rates. Eslam et al. discussed that prior studies [14–19] revealed no association of IFNL3 SNPs with SVR because patient numbers in those studies were too low to have the ability to detect any effect of IFNL3 SNPs on the outcome of treatment. In order to refute a lack of power due to high SVR rates in PP analysis a retrospective power analysis considering SVR ITT results was performed. Therefore, the number of included individuals in this study should be sufficient. However, despite the high number of patients included in this registry, neither in the PP nor in the ITT analysis an association between INFL3 SNPs and SVR could be observed. However, there was a linkage between IFNL3 SNPs and RVR and RVR remained the only independent factor associated with SVR in multivariate analysis. This was consistent with previous studies [23–28]. There are some limitations in our but also in the study by Eslam. Neither study is a prospective clinical trial. In Eslams study a fixed treatment duration of 24 weeks was implemented, whereas in this registry each participating physician could determine individual therapy durations, leading in general to an overtherapy like we published before [13]. Eslam et al., did not report the ribavirin doses actually used, which may impact the response in more difficult to treat patients. In our registry the recommendation were based on the German Clinical Practice Guideline (12–15 mg/kg) [23] and is often higher compared to the fixed 800mg dose initially suggested [30]. Our patients received 13.7 mg/kg in average. Our registry only included naïve patients while the study by Eslam et al., did not report if the patients were naïve or have been treated with PEG-IFN/RBV before [18]. Also the number of patients with moderate/severe fibrosis was higher in the study by Eslam. The association of IFNL3 and SVR may be lost in naïve easier to treat patients. The study by Eslam also included not only Caucasian patient.

Although there was no association between IFNL3 SNPs to SVR, a strong association to RVR was present and RVR remains the strongest factor for achieving SVR. In the end this may be even the more relevant association in the times of new emerging DAA therapies. In those “easy-to-treat” patients with low viral load, no signs of cirrhosis and the important milestone RVR, combination therapy of Peg-IFN and RBV may still be considered as first line treatment option. This concept is far less expensive [13] and could save resources for the more difficult to treat patients (cirrhosis, non-RVR) which should be treated with DAAs. We therefore suggest that the preferred patient populations for IFNL3 testing are naïve genotype 2 and especially naïve genotype 3 patients with low baseline viremia and without signs of cirrhosis. For example, G3 patients positive for IFNL3 CC genotype had 80% RVR of whom 94% achieved SVR in the PP analysis with a median of 24 weeks PEG-IFN/RBV.

In a recent study Susser et al. showed that IFN-L4 genotype was the best single predictor for SVR in genotype 3 infected patients [22]. However, we could not observe better predictive values for IFNL4 compared to IFNL3 in Genotype 3 patients. In the study of Susser et al. a high association of IFNL4 ss469415590 and IFNL3 rs12979860 was as well observed. Additionally, in their study they investigated only 191 patients with HCV genotype 3 which might influence results. Finally, the calculated p-value of 0.044 for the association of IFNL4 ss469415590 and SVR was only borderline significant.

Analysis of host genetic polymorphism usually requires EDTA blood. Sometimes, retrospective analyses of large cohorts are not possible due to the lack of stored samples. One of our other aims in this study was to assess if IFNL3 and IFNL4 genotyping was possible from serum samples. Our study revealed high concordance and in case of discordance results differed only by one base. Initially 712 samples had to be retested for IFNL3 genotyping with 687 cases (97%) revealing results for both SNPs. Thus, IFNL3 polymorphisms could easily be analysed from stored serum samples.

In conclusion, IFNL3 polymorphisms testing may be a tool for genotype 2/3 patients to decide if dual Peg-IFN/RBV therapy is an option to save resources in the times of new DAA therapies. IFNL4 showed similar predictive value in Caucasian patients and is not recommended in this setting.

## Supporting Information

S1 Data Collected data underlying our analyses (XLS)

S1 Fig SVR and RVR according to viral load and IFNL3 genotype. **(A)** SVR in patients with HCV genotype 3 with low and high viral load at baseline in relation to IFNL3 genotype, **(B)** RVR in patients with HCV genotype 2 with low and high viral load at baseline in relation to IFNL3 genotype, **(C)** SVR in patients with HCV genotype 2 with low and high viral load at baseline in relation to IFNL3 genotype.
(EPS)

S1 Table Clinical parameters at baseline for patients with preferable and non-preferable IFNL3 genotype. (DOC)

S1 Text In- and exclusion criteria. (DOCX)
