# Supplementary material for: Performance and Value of IFN-Lambda3 and IFN-Lambda4 Genotyping in Patients with Chronic Hepatitis C (CHC) Genotype 2/3 in a Real World Setting
Source: PLoS One. 2015 Dec 23;10(12):e0145622. doi: 10.1371/journal.pone.0145622 (PMC4689517; doi:10.1371/journal.pone.0145622)
Supplement: S1 Table — (DOC) [file pone.0145622.s003.doc]

**Table S3: clinical parameters at baseline for patients with preferable and non-preferable IFNL3 genotype**

| **Paramater** | **rs12979860** | | **p-value** | **rs8099917** | | **p-value** |
| --- | --- | --- | --- | --- | --- | --- |
|  | CC | non-CC |  | TT | non-TT |  |
| Age all [yrs]  median±SD  range  number | 44±11  22-73  n=281 | 44±10  22-82  n=404 | 0.5563 | 44±10  20-73  n=410 | 44±11  20-82  n=236 | 0.6692 |
| Age GT2 [yrs]  median±SD  range  number | 52±11  26-73  n=49 | 48±11  27-74  n=64 | 0.0954 | 51±12  26-73  n=73 | 48±11  29-74  n=38 | 0.4801 |
| Age GT3 [yrs]  median±SD  range  number | 42±10  21-70  n=228 | 43±10  22-82  n=334 | 0.7113 | 42±10  21-70  n=333 | 44±11  22-82  n=192 | 0.8308 |
| Male(%) all | 69 | 65 | 0.2561 | 69 | 62 | 0.0587 |
| Male (%) GT2 | 62 | 64 | 0.8208 | 62 | 63 | 0.9179 |
| Male (%) GT3 | 70 | 65 | 0.2161 | 70 | 61 | 0.0230 |
| BMI all [kg/m^2^]  median±SD  range  number | 24.7±4.8  15.2-47.9  n=275 | 25.2±4.4  17.2-45.9  n=395 | 0.6594 | 25.1±4.7  15.2-47.9  n=402 | 25.3±4.5  18.3-45.9  n=229 | 0.7942 |
| BMI GT2 [kg/m^2^]  median±SD  range  number | 26.3±4.8  16.7-44.7  n=48 | 25.7±4.9  18.3-42.6  n=63 | 0.7998 | 26.3±4.9  16.7-42.6  n=71 | 25.6±4.7  19.3-42.6  n=38 | 0.9594 |
| BMI GT3 [kg/m^2^]  median±SD  range  number | 24.5±4.8  15.2-47.9  n=224 | 25.1±4.3  17.2-45.9  n=326 | 0.4583 | 24.8±4.7  15.2-47.9  n=328 | 25.2±4.5  18.3-45.9  n=185 | 0.7449 |
| HCV-RNA all [log_10_IU/mL]  median±SD  range  number | 6.14±1.19  1.18-8.2  n=293 | 5.65±1.03  1.78-8.68  n=400 | 0.0292 | 6.03±1.17  1.18-8.68  n=428 | 5.59±1.00  2.21-8.21  n=229 | 0.0362 |
| HCV-RNA GT2  [log_10_IU/mL]  median±SD  range  number | 6.20±1.08  2.94-8.21  n=48 | 6.20±0.92  2.67-7.29  n=61 | 0.9962 | 6.18±1.05  2.67-7.54  n=71 | 6.37±0.85  4.50-8.21  n=36 | 0.1282 |
| HCV-RNA GT3  [log_10_IU/mL]  median±SD  range  number | 6.15±1.17  1.18-7.54  n=227 | 5.60±1.00  1.78-8.68  n=319 | 0.0179 | 6.02±1.13  1.18-8.68  n=332 | 5.52±1.00  2.21-7.40  n=182 | 0.0008 |
| SVR (%) all | 90 | 89 | 0.8679 | 90 | 88 | 0.4975 |
| SVR (%) GT2 | 97 | 93 | 0.4349 | 92 | 95 | 0.8364 |
| SVR (%) GT3 | 88 | 88 | 0.9224 | 90 | 86 | 0.3785 |
| RVR (%) all | 82 | 63 | <0.0001 | 80 | 55 | <0.0001 |
| RVR (%) GT2 | 86 | 71 | 0.0606 | 84 | 63 | 0.0162 |
| RVR (%) GT3 | 80 | 61 | <0.0001 | 79 | 53 | <0.0001 |
| Cirrhosis (%) all | 10.0 | 5.6 | 0.0289 | 7.9 | 4.9 | 0.1330 |
| Cirrhosis (%) GT2 | 10.0 | 1.6 | 0.0453 | 8.1 | 0 | 0.0712 |
| Cirrhosis (%) GT3 | 10.9 | 6.8 | 0.0894 | 8.6 | 6.3 | 0.3345 |
| Steatosis % all | 35.3 | 31.6 | 0.3468 | 36.9 | 27.7 | 0.0253 |
| Steatosis (%) GT2 | 28.3 | 37.5 | 0.3247 | 32.8 | 32.4 | 0.9610 |
| Steatosis (%) GT3 | 37.3 | 30.1 | 0.1077 | 38.0 | 26.2 | 0.0100 |
| **ALT all [U/L]**  median±SD  range  number | 110±123  13-1273  n=274 | 76±87  15-563  n=383 | <0.0001 | 100±114  13-1273  n=396 | 75±82  15-563  n=222 | <0.0001 |
| **ALT GT2 [U/L]**  median±SD  range  number | 77±102  14-542  n=47 | 65±77  16-353  n=61 | 0.4025 | 68±95  14-542  n=69 | 75±81  16-353  n=34 | 0.8400 |
| **ALT GT3 [U/L]**  median±SD  range  number | 114±125  13-1273  n=222 | 81±88  15-563  n=317 | <0.0001 | 109±115  13-1273  n=322 | 75±82  15-563  n=183 | <0.0001 |
| **AST all [U/L]**  median±SD  range  number | 70±92  13-949  n=265 | 55±54  14-384  n=370 | 0.0001 | 64±77  13-949  n=382 | 49±53  14-384  n=213 | 0.0007 |
| **AST GT2 [U/L]**  median±SD  range  number | 51±69  17-442  n=47 | 48±36  15-226  n=59 | 0.1306 | 48±63  16-442  n=69 | 44±31  15-127  n=32 | 0.1464 |
| **AST GT3 [U/L]**  median±SD  range  number | 76±97  13-949  n=213 | 58±56  14-384  n=306 | 0.0002 | 67±80  13-949  n=308 | 50±55  14-384  n=176 | 0.0010 |
